# Supplementary material for: Malaria prevention practices and associated factors among households of Hawassa City Administration, Southern Ethiopia, 2020
Source: PLoS One. 2021 May 13;16(5):e0250981. doi: 10.1371/journal.pone.0250981 (PMC8118284; doi:10.1371/journal.pone.0250981)
Supplement: S1 File — (DOCX) [file pone.0250981.s001.docx]

English version questionnaire

**Part I: Socio-demographic characteristics**

| **No** | **Questions** | **Coding Categories** | **Skip** |
| --- | --- | --- | --- |
|  | Sex of respondent | 1. Male 2. Female |  |
|  | Age (Incomplete years) | -___________________ |  |
|  | Address | 1. Urban 2. Rural |  |
|  | Marital status | 1.Single 2. Married  3. Divorced 4. Widowed |  |
|  | Religion | 1. Protestant 2. Orthodox  3. Catholic 4. Muslim  5. Other specify ____________ |  |
|  | Education | 1.Illitereate   1. Read & write 2. Elementary 3. High school 4. Diploma Or above |  |
|  | How much is the  household monthly income? | Monthly income in birr ______________ |  |
|  | Occupation | 1. House wife 2. Farmer 3. Government employee 4. Private employee 5. Others |  |
|  | Family size |  |  |

**Part 2: Questions related to the Knowledge of malaria**

| **No** | **Questions related to the Knowledge** | **Coding Categories** | **Skip** |
| --- | --- | --- | --- |
|  | Ever heard about malaria | 0. No  1. Yes |  |
|  | Cause of malaria mentioned | 1. Parasites  2. Bacteria  3. Viruses |  |
|  | Signs/symptoms of malaria mentioned | 1. Fevered 2. chills 3. Headache 4. Joint pain 5. Vomiting 6. Others |  |
|  | When does a mosquito bite mostly? | 0. Day  1. Night |  |
|  | Common breeding sites | 0. Dry area  1. Water body |  |
|  | Common resting sites | 1. House 2. Outside house |  |
|  | Mode of transmission | 1. Mosquito bite 2. Fly bite 3. Drinking water |  |
|  | Preventive methods mentioned (any) | 1. ITN use 2. Drainage 3. Covering body 4. Smoke 5. Repellant use 6. Close openings |  |
|  | Advantage of mosquito nets | 1. Prevent mosquito bite 2. Attract mosquito 3. Group most affected by malaria 4. Pregnant & children 5. Other groups |  |

**Part 3: Questions related to the attitude towards malaria**

| ***Level of agreement of Attitude towards malaria prevention measures utilization*** | | ***Strongly Disagree (1)*** | ***Disagree (2)*** | ***Neutral (3)*** | ***Agree (4)*** | ***Strongly Agree (5)*** |
| --- | --- | --- | --- | --- | --- | --- |
|  | I think that Malaria is a life-threatening disease |  |  |  |  |  |
|  | I think that Malaria is a communicable disease |  |  |  |  |  |
|  | I think the best way to prevent myself getting Malaria is to avoid getting mosquito bites |  |  |  |  |  |
|  | I am sure that anyone can get Malaria |  |  |  |  |  |
|  | I believe sleeping under a mosquito net during the night is one way to prevent myself getting Malaria |  |  |  |  |  |
|  | I am sure that self-treatment may dangers my health |  |  |  |  |  |
|  | In my opinion, children and pregnant women are at higher risk of Malaria |  |  |  |  |  |
|  | I think that one can’t recover spontaneously from Malaria without any treatment |  |  |  |  |  |
|  | I think that malaria can’t transmit through contact |  |  |  |  |  |
|  | I might be at a greater risk of getting Malaria if I work and sleep overnight in the outside |  |  |  |  |  |
|  | I think that it is dangerous when Malaria medicine is not taken completely |  |  |  |  |  |
|  | I can buy anti-Malaria drugs from the drug shop/pharmacy to treat myself when I get Malaria |  |  |  |  |  |
|  | I think that I should have blood test if I have fever |  |  |  |  |  |
|  | I will seek for advice I get Malaria |  |  |  |  |  |
|  | In my opinion, it is very important to check for an expiry date of the drug before taking it |  |  |  |  |  |

**Part 4: Questions on Other related factors**

| **No** | **Questions** | **Coding Categories** | **Skip** |
| --- | --- | --- | --- |
|  | Perceived quality of care at nearest facility | 1. Very bad 2. Bad 3. Fair 4. Good 5. Excellent |  |
|  | If health facility, how good was the care? | 1. Very bad 2. Bad 3. Fair 4. Good 5. Excellent |  |
|  | Any Other barrier for utilization of the Insecticide Spraying and Mosquito Nets | 1. Inadequate resources 2. unavailability of trained staff, 3. poor supervision and mentorship of staff, 4. personnel turnover in government 5. Visits from the community health worker 6. Use of traditional preventive approaches |  |

**Part 5: Questions related to Practice of malaria prevention measures**

| ***Frequency of malaria prevention measures utilization*** | | ***Never (0)*** | ***Sometimes (1)*** | ***Always (2)*** |
| --- | --- | --- | --- | --- |
|  | How often do you sleep in a mosquito net? |  |  |  |
|  | How often do other members of the household sleep in mosquito nets? |  |  |  |
|  | How often do you use mosquito repellents in your house? |  |  |  |
|  | How often do you use anti-mosquito spray in your house? |  |  |  |
|  | How often your house is sprayed with anti-mosquito chemical spray (IRS) by community health workers? |  |  |  |
|  | How often do you clean/cut bushes around your house? |  |  |  |
|  | How often do you clean stagnant water near your house? |  |  |  |
|  | How often do you visit the health center when you fall sick? |  |  |  |
|  | How often do you receive visits from the community health worker? |  |  |  |
|  | How often do you participate in malaria prevention campaigns? |  |  |  |
