# Supplementary material for: Malaria prevention practices and associated factors among households of Hawassa City Administration, Southern Ethiopia, 2020
Source: PLoS One. 2021 May 13;16(5):e0250981. doi: 10.1371/journal.pone.0250981 (PMC8118284; doi:10.1371/journal.pone.0250981)
Supplement: Attachment — Submitted filename: 2. Point-by-point response to reviewers file.docx [file pone.0250981.s003.docx]

29 March, 2021

Dear respected reviewers,

***Ref: A point-by-point response to the comments***

**Dear reviewer 1**, First of all I would like to thank you very much for your appreciation and forwarding constructive and insightful comments which we believe that it would improve the quality of our manuscript. Thus, according to your comments, suggestions and questions the following amendments has been taken.

**Amendments done on the minor comments raised by reviewer-1**

1. **INTRODUCTION**: Update data on Malaria burden with Global Malaria report 2020.

**Answer:** I have updated the burden of malaria based on Global Malaria report 2020

Furthermore, add some sentence on why also during COVID 19 pandemic is important Malaria control (see and cite Malaria and COVID-19: Common and Different Findings. Trop Med Infect Dis. 2020 Sep 6;5(3):141. doi: 10.3390/tropicalmed5030141. )

**Answer:** I have downloaded the aforementioned article and included in our reference list after extracting valuable data’s.

2. **Discussion:** add some sentence on COVID-19 to contextualize your paper

**Answer:** I have added a sentence describing the existence of COVID-19 and malaria based on your suggestion.

**Amendments done on the comments raised by reviewer-2**

**Dear Reviewer 2:** It is my pleasure to extend my earnest gratitude for your mindful and deep comments and suggestions given to our manuscript. We highly believe that your comments have a big share in the improvement of our manuscript. Here are the amendments taken based on your comments.

1. Abstract

Background: line 1 should read ‘’Despite it is easily preventable, malaria still remains a major…..’’

**Answer:** Corrected

2. Introduction:

Page 2 Line 1 should read ‘’have been reported, of these, nearly 69.2%...’’

**Answer:** Corrected

Page 2 Line 4/5 ‘’in areas below 2,000 meters’’ 2,000 meters below what? this is not clear, clarify

**Answer:** ’in areas below 2,000 meters altitude’’

Line 10 ‘’WHO is highly recommends’’ corrected needed here

**Answer:** Corrected

3. Methods section

Data Processing and analysis: 3rd sentence says ‘’the data were presented by tables, graphs and different interactive charts’’ but you only have tables in your manuscript

**Answer:**  ‘’the data were presented by tables, graphs and different interactive charts’’ changed to …”the data were presented by tables”.

4. **Results**

Your result reporting includes both a narrative (running commentary) of the findings and tables……. give highlight/summary of the result for each table then refer readers to the appropriate table

**Answer:**  Based on your suggestions I have given highlight of the result for each table.

**Knowledge about malaria prevention among study participants: page** 8

Line 6 ‘…participants were knew’’ should be ‘participants knew’.

**Answer:**  Corrected!!

Line 7 ‘’571 (97.8%) were ever used’’ should be ‘ever used’

**Answer:**  Corrected!!

Line 8: ‘‘Regarding to the’’ should be ‘regarding the’

**Answer:**  Corrected!!

**Attitude towards Malaria prevention and control among households: page 9**

Please recast as too many use of the word WERE renders this statement meaningless. This also applies to the rest of your results where you used the word WERE a lot, it makes what you are writing difficult to understand

**Answer:**  Corrected!!

**Discussion**: it should be discussion, but you have written ‘discussions’

**Answer:**  discussions changed in to ‘discussion’

**Reference:** I suggest authors look up a similar study and add to their references

**Answer:**  I have added the suggested article in our reference list.
